# Anti‑epidermal growth factor receptor monoclonal antibody therapy in locally advanced head and neck cancer: A systematic review of phase III clinical trials

**DOI:** 10.3892/mi.2024.165

**Published:** 2024-05-29

**Authors:** Lekha Madhavan Nair, Rejnish Ravikumar, Malu Rafi, Jissy Vijo Poulose, Nijo Jose, Krishnapriya Pisharody, Kainickal Cessal Thommachan

**Affiliations:** 1 Department of Radiation Oncology, Regional Cancer Centre, Thiruvananthapuram, Kerala 695011, India; 2 Department of Palliative Medicine, DEAN Foundation Hospice and Palliative Care Centre, Chennai, Tamil Nadu 600010, India

**Keywords:** locally advanced head and neck squamous cell carcinoma, anti-EGFR antibody, panitumumab, cetuximab, nimotuzumab, zalutumumab

## Abstract

The present systematic review evaluated the effectiveness of anti-EGFR therapy in combination with radiotherapy (RT) or with chemoradiation compared with the existing standard of care for the treatment of locally advanced head and neck squamous cell carcinoma (LAHNSCC). The PubMed, SCOPUS, EMBASE and COCHRANE databases were searched and 12 phase III randomized controlled trials were included. The effectiveness of the anti-EGFR monoclonal antibody cetuximab was evaluated in nine trials. Nimotuzumab (one trial), zalutumumab (one trial) and panitumumab (one trial) were the monoclonal antibodies evaluated in the remaining three trials. One study tested the effectiveness of adding cetuximab to radical RT and found that patients with LAHNSCC exhibited improvement in locoregional control (LRC), overall survival (OS) and progression-free survival (PFS) compared with those of patients treated with RT alone. A total of three studies tested the effectiveness of adding an anti-EGFR agent to chemoradiation. Of these, a single institution study in which patients received cisplatin at 30 mg/m^2^ weekly, instead of the standard doses of 100 mg/m^2^ every 3 weeks or 40 mg/m^2^ every week, reported significant improvement in PFS with the addition of nimotuzumab to chemoradiotherapy without an improvement in overall survival. However, the other two studies indicated that, when added to standard chemoradiation, the anti-EGFR monoclonal antibodies cetuximab or zalutumumab did not improve survival outcomes. Two phase III trials evaluated RT plus an anti-EGFR agent compared with chemoradiation alone. Of these, one study reported inferior outcomes with cetuximab-RT in terms of OS and LRC, whereas the other study with panitumumab plus RT failed to prove the non-inferiority. Two trials evaluated induction chemotherapy followed by cetuximab-RT compared with chemoradiotherapy and reported no benefits in terms of OS or PFS. Furthermore, one study evaluated induction chemotherapy followed by cetuximab-RT compared with induction chemotherapy followed by chemoradiotherapy and found no improvement in OS or PFS. Finally, three phase III trials tested the effectiveness of cetuximab plus RT in the treatment of human papillomavirus-positive oropharyngeal carcinoma, and found it to be inferior compared with cisplatin-RT in terms of OS, PFS and failure-free survival. Based on the aforementioned findings, it is difficult to conclude that anti-EGFR therapy in any form has an advantage over conventional chemoradiation in the treatment of LAHNSCC.

## Introduction

Head and neck squamous cell carcinoma (HNSCC) is reported as the seventh most common type of cancer worldwide (1). In total, >50% of patients with head and neck cancer present with loco-regionally advanced disease at the time of diagnosis (2). The standard treatment for locally advanced head and neck squamous cell carcinoma (LAHNSCC) is concurrent chemoradiation or surgery followed by adjuvant treatment (3,4). The MACH-NC meta-analyses had shown an overall survival benefit with concurrent chemotherapy compared to radiotherapy (RT) alone (5-7). However, the combined modality treatment is associated with higher rates of acute and late toxicities (8). Even following combined modality treatment, ~50% of patients experience relapse (9). Therefore, to improve treatment efficacy and prevent the incidence of adverse events, novel small molecules designed to target various different signaling pathways are increasingly being tested alongside RT or chemoradiation (9-13).

**Figure 1:**
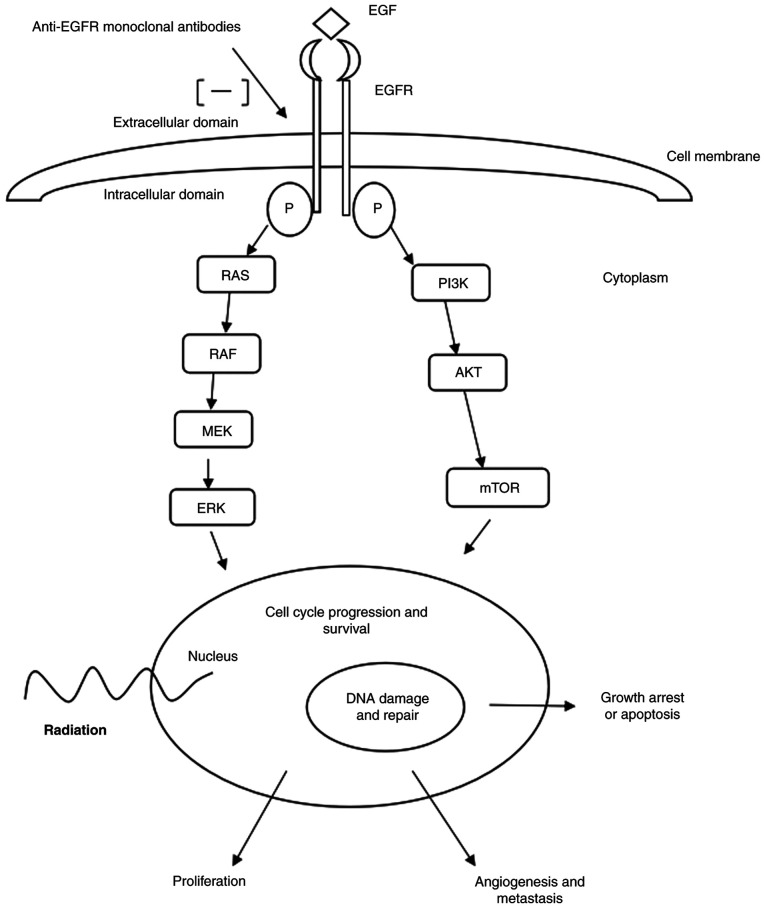
Diagram of the EGFR pathway and mechanism of action of anti-EGFR monoclonal antibodies. p, phosphorylated. -, inhibition of the pathway.

In total, 80-90% of HNSCC cases are reported to exhibit upregulated expression levels of EGFRs. EGFR upregulation in cancer cells is associated with local treatment failure, resistance to radiation, increased incidence of distant metastases and decreased survival. In cancer cells with upregulated EGFR expression, radiation can activate the EGFR pathway, resulting in re-proliferation of tumor cells and DNA repair, resulting in treatment failure. Anti-EGFR monoclonal antibodies block the activation of the EGFR pathway, resulting in inhibition of tumor cell proliferation, tumor angiogenesis and the development of metastasis. Anti-EGFR monoclonal antibodies also play a role in the enhancement of the effect of radiation through suppression of DNA repair during RT (10-20). Thus, improved disease control is considered to be achieved through the synergistic action of radiation and EGFR blockade. Fig. 1 demonstrates the EGFR pathway by which EGFR blockade is considered to work in the treatment of LAHNSCC.

The objective of the present systematic review was to gather evidence from a list of published phase III clinical trials that investigated the efficacy of anti-EGFR monoclonal antibody therapy for the definitive treatment of LAHNSCC, including human papillomavirus (HPV)^+^-oropharyngeal carcinoma (OPC). Ultimately, the present study aimed to compare the effectiveness of anti-EGFR therapy in combination with RT or chemoradiotherapy with that of the non-surgical standard treatment method for the treatment of LAHNSCC.

## Materials and methods

### Inclusion/exclusion criteria

The inclusion criteria were as follows: i) Articles published in peer-reviewed journals; ii) phase III randomized clinical trials (RCTs); iii) patients with LAHNSCC and patients with HPV^+^-OPC; iv) patients that received an anti-EGFR agent in combination with RT or chemoradiotherapy; and v) patients that received non-surgical standard treatment (RT or chemoradiotherapy). The exclusion criteria were as follows: i) Phase I or phase II clinical trials; ii) observational studies; and iii) studies involving patients with nasopharyngeal carcinoma.

### Data sources and literature search

The present study was conducted based on the Preferred Reporting Items for Systematic Reviews and Meta-Analyses (PRISMA) guidelines (21). The PubMed (www.ncbi.nlm.nih.gov/pubmed/), SCOPUS (https://www.scopus.com/), EMBASE (https://www.embase.com/) and COCHRANE (https://www.cochranelibrary.com/) databases were searched without any language or date limits. Searches were performed using the following key words: ‘head and neck squamous cell carcinoma’, ‘head and neck cancer’, ‘locally advanced’, ‘anti-EGFR antibody’, ‘panitumumab’, ‘cetuximab’, ‘nimotuzumab’ and ‘zalutumumab’. A sensitive search filter was used to identify RCTs. The original literature search was conducted in November 2022, and this was later updated in January 2024.

### Study selection

Titles from the search results were exported to Endnote (https://www.myendnoteweb.com/EndNoteWeb.html) by one author before any duplicates were removed. Titles pertaining to phase I/II studies, immunotherapy trials or trials involving patients with recurrent/metastatic HNSCC were all removed by the same author. The remaining titles were then screened further for relevance. Abstracts of potentially eligible studies were independently analyzed by two authors and articles pertaining to eligible studies were selected for full-text review. Disagreements were resolved through discussion among the authors at each step. Phase III RCTs that evaluated the role of an anti-EGFR agent in combination with RT or chemoradiotherapy were included in the present systematic review. Cetuximab, nimotuzumab, panitumumab and zalutumumab were the four anti-EGFR agents studied. The following data were extracted from each study: Patient population, intervention, parameters being compared, outcomes and adverse events. The search syntax is provided in Table SI, Table SII, Table SIII and Table SIV.

## Results

### Description of included studies

**Figure 2:**
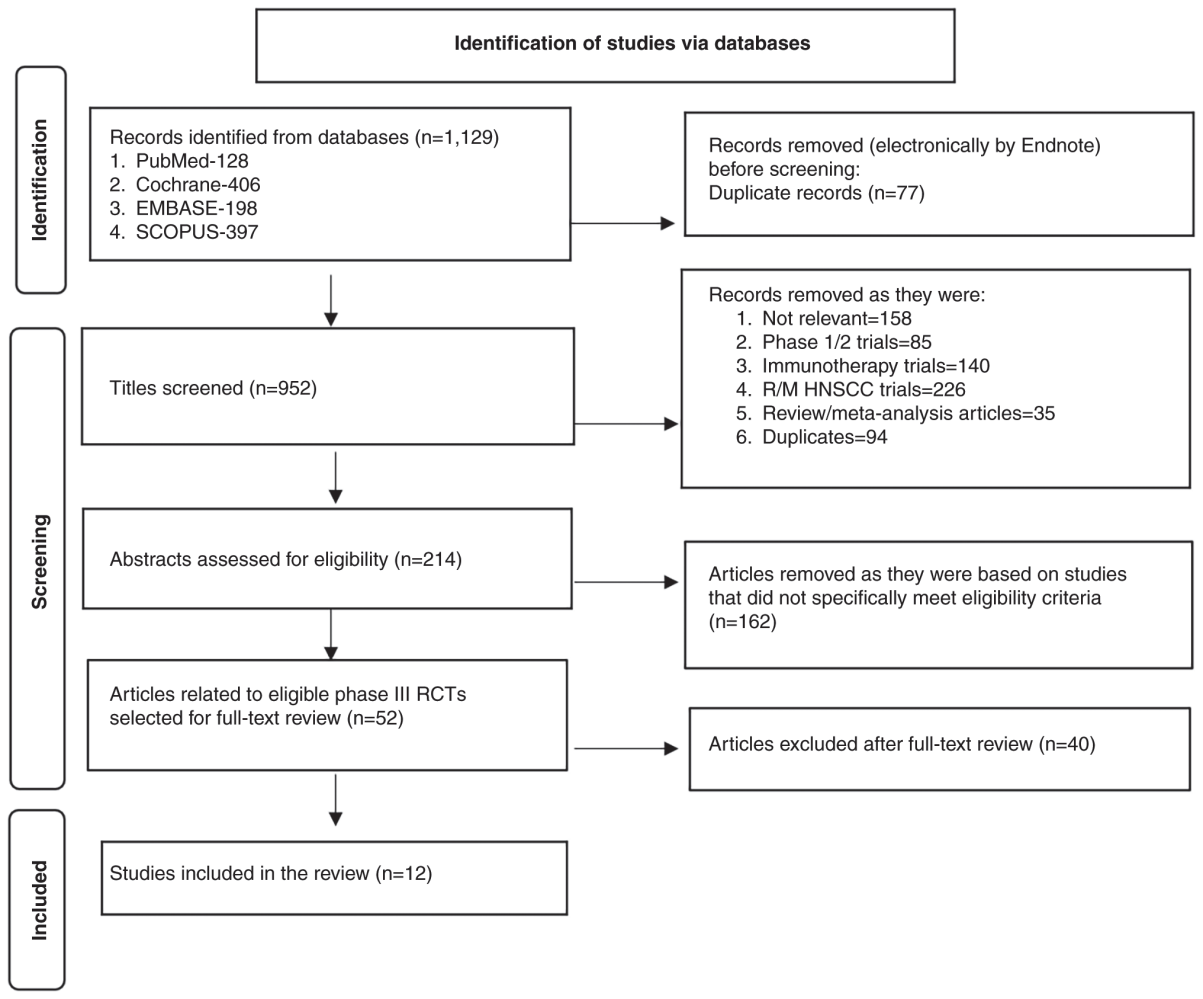
Preferred Reporting Items for Systematic Reviews and Meta-Analyses flow diagram of article selection. HNSCC, head and neck squamous cell carcinoma; RCT, randomized clinical trial; R/M, recurrent/metastatic.

The updated literature search performed in January, 2024 identified 1,129 titles from the four databases. After removing duplicates, 952 titles were screened, among which 214 titles were selected for abstract review to identify potentially eligible studies. After abstract review, 162 records were excluded, and the remaining 52 articles were selected for full-text review. From these papers, 12 original articles on 12 unique phase III clinical studies were selected for the present review. The PRISMA flow chart of study selection for the present review is shown in Fig. 2. A detailed summary of the 12 RCTs (22-33) that tested the effectiveness of anti-EGFR monoclonal antibodies for the treatment of LAHNSCC is provided in Table I.

All trials included patients >18 years of age. Whilst the majority of the trials included patients with malignancies originating from the oropharynx, hypopharynx, larynx and oral cavity, in two of the studies (22,23), the oral cavity subsite was not included. In total, three studies (31-33) compared the efficacy of cetuximab + RT against cisplatin + RT for HPV^+^-OPC. The majority of the studies used The American Joint Committee on Cancer (AJCC)/Union for International Cancer Control 7th edition staging system (34), except for the trial performed by Bonner *et al* (22), which used the AJCC 1998 system (35) and the RTOG 0522 trial (23), which used the AJCC 6th edition guidelines (36).

Only one study (22) compared the effectiveness of an anti-EGFR agent with that of RT alone, whilst three studies (23-25) assessed the potential benefit of adding an anti-EGFR agent into the chemoradiotherapy regimen compared with concurrent chemoradiotherapy alone. In two of the included studies (26,27), patients in the treatment group received an anti-EGFR agent + RT, whilst patients in the control group received chemoradiotherapy alone. In two other studies (28,29), patients in the intervention group received induction chemotherapy prior to treatment with an anti-EGFR agent + RT, whereas patients in the control group received chemoradiotherapy alone. In one study (30), patients who responded to induction chemotherapy were randomized to evaluate the effectiveness of the anti-EGFR agent + RT compared with that of cisplatin + RT. In three studies (31-33), the efficacy of cetuximab + RT was evaluated compared with that of cisplatin + RT in HPV^+^-OPC.

Cetuximab was the anti-EGFR agent evaluated in nine of the studies (22,23,26,28-33). Nimotuzumab, panitumumab and zalutumumab were the anti-EGFR monoclonal antibodies evaluated in three studies (24,25,27). Docetaxel+ cisplatin+ fluorouracil (TPF) induction chemotherapy was given to the intervention arm before cetuximab + RT in two studies (28,29), whilst in one study (29), TPF induction therapy was given in both the control and intervention arms. In one study accelerated RT was used both in the intervention arm and the control arm (25), while in another study the intervention arm received accelerated RT and control arm received conventional RT (27). In all other trials, conventional RT was used in both the control and intervention arms. Chemoradiotherapy was the standard treatment strategy used in the control arm in all but one study (22), in which RT alone was given as the standard therapeutic method. Regarding concurrent chemotherapy, cisplatin administered three times per week was used in six trials (23,27,29-32), whilst weekly cisplatin was used in four trials (24,25,26,33) and one trial used carboplatin + 5-flurouracil for concurrent chemotherapy (28). The radiation dose was in the 60-70 Gy range in these studies. While the phase III trial performed by Bonner *et al* (22) used two dimensional-RT, in total, four trials used intensity-modulated RT (IMRT) or three-dimensional conformal RT (3DCRT) techniques for radiation planning and delivery (23,27-29). By contrast, four trials used IMRT for radiation planning and delivery for all patients. (25,31-33). In the trial by Patil *et al* (24), 2D-RT, 3DCRT or IMRT was used. In addition, one study (26) used IMRT, helical tomotherapy or volumetric arc therapy for radiation planning. The trial by Hitt *et al* (30) used the 3DCRT technique for all patients.

### Outcomes

Of the 12 trials included in the present review, four studies (23,24,27,28) assessed progression-free survival (PFS) as the primary endpoint, whilst four studies (26,29,30,32) assessed OS as the primary endpoint. Locoregional control (LRC) was the primary outcome in two studies (22,25). Overall severe toxicity was assessed as the primary outcome in the De-ESCALaTE trial by Mehanna *et al* (31). Symptom severity as assessed using the MD Anderson Symptom Inventory Head and Neck Symptom Severity Scale (MDASI-HN) was the primary endpoint in the TROG 12.01 trial (33,37).

Since the studies were heterogeneous in terms of treatment combinations, for the purpose of cataloging the evidence, the studies were categorized into six groups based on the nature of the treatment combinations involved. The groups were as follows: i) RT + concurrent anti-EGFR monoclonal antibody vs. RT alone; ii) Concurrent chemoradiotherapy + anti-EGFR monoclonal antibody vs. concurrent chemoradiotherapy alone; iii) RT + anti-EGFR monoclonal antibody vs. concurrent chemoradiotherapy alone; iv) Three-drug induction chemotherapy followed by anti-EGFR monoclonal antibody + RT vs. chemoradiotherapy alone; v) induction chemotherapy followed by RT + anti-EGFR monoclonal antibody vs. induction chemotherapy followed by chemoradiotherapy; and vi) Anti-EGFR monoclonal antibody in HPV^+^-oropharyngeal cancer (OPC).

### RT + concurrent anti-EGFR monoclonal antibody vs. RT alone (1 RCT)

In a phase III clinical trial, Bonner *et al* (22) randomized patients (n=424) with stage III or IV, non-metastatic, squamous cell carcinoma of the oropharynx, hypopharynx, or larynx to receive either RT + cetuximab (intervention arm) or RT alone (control arm). Cetuximab was administered 1 week before RT at a dose of 400 mg/m^2^, followed by 250 mg/m^2^ weekly for the duration of RT. The primary endpoint was the duration of LRC. The secondary endpoints were OS, PFS, overall response rate (ORR) and safety. The duration of LRC was 24.4 months in the intervention group, which received cetuximab + RT, compared with 14.9 months in the control group, which received RT alone [hazard ratio (HR) for locoregional progression, 0.68; 95% CI, 0.52-0.89; P=0.005]. The median PFS was 17.1 months for patients who received RT + cetuximab whereas it was 12.4 months for patients receiving RT alone (HR for disease progression/death, 0.70; 95% CI, 0.54-0.90; P=0.006). The ORR was 74% in the intervention group, compared with 64% in the control group (odds ratio, 0.57; 95% CI, 0.36-0.90; P=0.02). After a median follow-up duration of 54 months, the median OS was 49 months in the intervention arm compared with 29.3 months in the control arm (HR for mortality, 0.74; 95% CI, 0.57-0.97; P=0.03) (38). In terms of adverse events, apart from an increased incidence of acneiform rash and infusion reactions, the two groups did not show any statistically significant differences in terms of grade ≥3 toxicities. The most frequently observed side effects of RT included mucositis, dysphagia, pain, xerostomia, weight loss and performance status deterioration, the incidences of which were similar in the two groups. Severe late effects associated with RT were also similar in the two groups. The 5-year OS was found to be 45.6% in the cetuximab + RT group and 36.4% in the RT-alone control group (38). In addition, the 5-year OS was significantly higher among patients who experienced cetuximab-induced acneiform rash of grade ≥2 severity compared with those who had no rash or a grade1 rash (HR, 0.49; 95% CI, 0.34-0.72; P=0.002). This finding supported the possibility of cetuximab induced acneiform rash being a biomarker of an immunological response associated with optimal outcomes (38,39). In conclusion, this trial demonstrated the effectiveness of cetuximab in combination with RT for improving LRC whilst reducing mortality compared with RT alone (22,38).

### Concurrent chemoradiotherapy + anti-EGFR monoclonal antibody vs. concurrent chemoradiotherapy alone (3 RCTs)

In total, three phase III clinical trials (23-25) tested the effectiveness of concurrent chemoradiotherapy + an anti-EGFR agent compared with concurrent chemoradiotherapy alone. In the RTOG 0522 trial, 891 patients with stage III or IV non-metastatic squamous cell carcinoma of the oropharynx, hypopharynx or larynx were randomly allocated to receive RT + concurrent cisplatin with or without cetuximab (23). In this trial, PFS was the primary outcome. Secondary outcomes included OS, locoregional failure (LRF), distant metastasis and adverse events. The 3-year PFS was 58.9% (95% CI, 54.2-63.6%) in the intervention arm (cetuximab + cisplatin-RT) vs. 61.2% (95% CI, 56.7-65.8%) in the control arm (cisplatin-RT; P=0.76). The 3-year OS was 75.8% (95% CI, 71.7-79.9%) in the intervention arm vs. 72.9% (95% CI, 68.7-77.1%) in the control arm (P=0.32). The 3-year LRF was 25.9% (95% CI, 21.7-30.1%) in the intervention arm compared with 19.9% (95% CI, 16.2-23.7%) in the control arm (P=0.97). The rates of distant metastasis in the two arms were not significantly different (13.0 vs. 9.7%; P=0.08). Updated results after a median follow-up duration of >10 years suggested that the addition of cetuximab to RT and cisplatin did not improve the PFS or OS or prevented distant metastasis (40). No significant differences were found between the intervention and control arms in terms of the 30-day mortality rate (1.8 vs. 2.0%; P=0.81). In terms of adverse events, patients who received cetuximab + cisplatin and RT experienced more treatment-related grade 5 adverse events (10 vs. 3; P=0.05) compared with patients in the control group. The incidence of grade 3-4 radiation mucositis was found to be higher in the cetuximab arm compared with the control arm (43.2 vs. 33.3%; P=0.003). Patients in the intervention group exhibited higher rates of grade 3 to grade 4 skin reactions, fatigue, anorexia, and hypokalemia during the first 90 days of therapy compared with those of the control group. Therefore, the findings of this trial suggested the absence of benefits of adding cetuximab to cisplatin + RT in terms of PFS or OS.

A single institution study (n=536) by Patil *et al* (24) included patients with non-metastatic, stage III or IV squamous cell carcinoma of the oropharynx, larynx, hypopharynx or oral cavity who were randomly allocated to receive RT + cisplatin (30 mg/m^2^) with or without nimotuzumab (24). PFS was the primary outcome in this trial and the median follow-up duration was 39.13 months. Secondary outcomes included LRC, disease-free survival (DFS), OS, and adverse events. The 2-year PFS was 61.8% (95% CI, 55.2-67.7) in the intervention arm, compared with 50.1% (95% CI, 43.7-56.2) in the control arm (HR, 0.69; 95% CI, 0.53-0.89; P=0.004). The 2-year LRC was 67.5% (95% CI, 60.9-73.3%) in the intervention arm vs. 57.6% (95% CI, 50.9-63.6%) in the control arm (HR, 0.67; 95% CI, 0.50-0.89; P=0.006). The 2-year OS was 63.8% (95% CI, 57.3-69.6%) in the intervention arm vs. 57.7% (95% CI, 50.9-63.6%) in the control arm (HR, 0.84; 95% CI, 0.65-1.08; P=0.163). DFS was also improved with the addition of nimotuzumab (HR, 0.71; 95% CI, 0.55-0.92; P=0.008). In terms of adverse events, a higher incidence of grade 3-5 mucositis occurred in the nimotuzumab + cisplatin + RT arm (66.7 vs. 55.8%; P=0.01). However, the incidence of other grade 3-5 adverse effects was similar between the two groups. Therefore, the results of this trial suggested that nimotuzumab improved both LRC and DFS when it was added to the RT + weekly 30 mg/m^2^ cisplatin regimen, albeit without improvements in OS.

The DAHANCA 19 study included patients (n=619) with biopsy-verified HNSCC of the oral cavity, oropharynx, hypopharynx, and larynx in which the investigators tested the efficacy of the anti-EGFR agent zalutumumab (25). Patients in the intervention group received accelerated RT + nimorazole with zalutumumab, whilst the control group received accelerated RT + nimorazole only. Patients in both arms of the study received 40 mg/m^2^ cisplatin weekly. The 3-year LRC rates were similar between the intervention (accelerated RT + nimorazole + zalutumumab) and control (accelerated RT + nimorazole) arms (78 vs. 79%; HR, 0.8; 95% CI, 0.6-1.2). Disease-specific survival (DSS; HR, 1.0; 95% CI, 0.7-1.7) and OS (HR, 0.9; 95% CI, 0.6-1.3) were also similar for the intervention and control arms. The outcomes were not influenced by p16 positivity (HR, 1; 95% CI, 0.6-1.8) or p16 negativity (HR, 0.8; 95% CI, 0.5-1.4). The 5-year LRC rates were also similar between the intervention arm and the control arm (70 vs. 74%; HR, 1.10; 95% CI, 0.81-1.50). There was no significant impact on the 5-year DSS (HR, 1.12; 95% CI, 0.79-1.60) or OS (HR, 1.17; 95% CI, 0.89-1.52) (41). In addition, 94% of the patients in the zalutumumab arm experienced a skin rash. Grade 3-4 skin rash occurred in 29% of patients, where 13% of patients had to terminate the zalutumumab treatment due to the rash. Patients in the zalutumumab arm experienced significant rates of confluent mucositis (70% vs. 56%, P=0.001) and grade 3-4 in-field skin reaction (27% vs. 4% P<0.0001) (41).

Therefore, this trial concluded that zalutumumab, when added to chemoradiotherapy, did not improve outcomes like LRC or OS and therefore not an effective treatment option for patients with LAHNSCC.

### RT + anti-EGFR monoclonal antibody vs. concurrent chemoradiotherapy alone (2 RCTs)

There were two phase III RCTs that compared the efficacy of RT + anti-EGFR monoclonal antibodies with that of chemoradiotherapy alone (26,27). The ARTSCAN III trial included patients with stage III and IV SCC of the oropharynx, hypopharynx, or larynx without distant metastases (26). Patients were randomly allocated to receive either cetuximab + RT or cisplatin + RT. The primary endpoint set for the ARTSCAN III trial was OS, whereas the secondary endpoints included LRC, local control with dose-escalated RT, distant failure and adverse events. After 3 years, the OS was 78% (95% CI, 71-85%) in the cetuximab RT arm, whereas it was 88% (95% CI, 83-94%) in the cisplatin + RT group (HR, 1.63; 95% CI, 0.93-2.86; P=0.086). The cumulative incidence of locoregional failures at 3 years was 23% (95% CI, 16-31%) in the intervention arm vs. 9% (95% CI, 4-14%) in the control arm (adjusted cause-specific HR, 2.49; 95% CI, 1.33-4.66; P=0.0045) There was no difference in the cumulative incidence of distant failures. Post hoc subgroup analyses revealed that the HR for OS was 5.70 (95% CI, 1.67-19.5) for patients with p16^+^-OPC treated with RT + cetuximab compared with those treated with RT + cisplatin (P=0.03). By contrast, the acute toxicity profiles differed between the intervention and control arms. The incidence of acute mucositis (P=0.035), skin reactions (P=0.001) and acneiform rashes (P<0.001) was higher in the cetuximab group compared with the cisplatin group whereas the incidence of nausea (P=0.001), vomiting (P=0.015), acute kidney injury (P<0.001), tinnitus (P=0.002), dysphagia (P=0.033) and neutropenia (P<0.001) was significantly higher in the cisplatin arm. Late toxicities, such as taste alteration and hearing impairments, were significantly more common in the cisplatin arm, whilst late pain and mucosal toxicities were more common in the cetuximab arm. Based on the findings of this trial, cetuximab + RT was deemed to be inferior to cisplatin + RT for the treatment of LAHNSCC.

In the HN.6 trial, 320 patients with locoregionally advanced squamous cell carcinoma of the oral cavity, oropharynx, larynx, or hypopharynx were randomly allocated to receive standard fractionation RT and concurrent cisplatin (arm A) or accelerated RT and concurrent panitumumab (arm B) (27). In this trial, the primary endpoint was PFS. It was designed as a non-inferiority trial (a test of non-inferiority of arm B to arm A was to be done if superiority of arm B was not detected in the primary analysis and non-inferiority would be claimed if the upper limit of a two-sided 95% CI for HR was ≤1.15). There were no statistically significant differences in the PFS between the two arms (HR, 0.95; 95% CI, 0.60-1.50; stratified log-rank test, P=0.83). The 2-year PFS was 73% (95% CI, 65-79%) for arm A and 76% (95% CI, 68-82%) for arm B. Regarding OS, there was no statistically significant difference between the two arms (HR, 0.89; 95% CI, 0.54-1.48; stratified log-rank test, P=0.66). The 2-year OS was 85% (95% CI, 78-90%) for arm A, compared with 88% (95% CI, 82-92%) for arm B. There were no statistically significant differences between the two arms in terms of the 2-year cumulative incidence of local recurrence, regional recurrence, or distant recurrence. Sub-group analyses based on performance status, T category, N status, primary site, p16 expression status and smoking history did not reveal any differences in PFS between the two arms. There was also no significant difference in the quality of life (QoL) parameters (measured using Functional Assessment Cancer Therapy-Head &Neck) or swallowing outcomes between the two arms (27,42). At 1 year post treatment no difference was found between the arms in terms of FACT-H&N change from baseline: -1.70 (control arm) and-4.81 (intervention arm), P=0.194. Swallowing related QOL (measured using SWAL-QOL and MDADI) declined from baseline to every subsequent time point (42).

Regarding adverse events, ototoxic effects (such as hearing loss and tinnitus), gastrointestinal tract symptoms (such as nausea, vomiting and dehydration), nephrotoxic effects and weight loss were more common in the cisplatin + standard RT arm. By contrast, skin toxicity and grade ≥3 mucositis were more common in the accelerated RT + panitumumab group. The incidence of non-hematological grade ≥3 adverse events was similar between the two arms (88% in arm A vs. 92% in arm B; P=0.25). These results suggested that the trial failed to prove the non-inferiority of panitumumab + accelerated RT in terms of PFS or OS compared with standard chemoradiotherapy for the treatment of LAHNSCC.

### Three-drug induction chemotherapy followed by anti-EGFR monoclonal antibody + RT vs. chemoradiotherapy alone (2 RCTs)

GORTEC 2007-02(28) and INTERCEPTOR-GONO (29) are two phase III clinical trials that investigated the effectiveness of a three-drug induction chemotherapy followed by RT + an anti-EGFR agent compared with that of chemoradiotherapy alone. GORTEC 2007-02 included patients with stage III-IV non-metastatic SCC of the oral cavity, oropharynx, hypopharynx, or larynx, with heavy nodal disease (N2b, N2c or N3), which are known to develop distant metastasis (28,43). The intervention arm received 3 cycles of TPF followed by cetuximab + RT (n=181). The control arm received 3 cycles of concurrent chemotherapy (carboplatin 70 mg/m^2^/day on days 1-4; and fluorouracil 600 mg/m^2^/day on days 1-4, continuous infusion every 3 weeks). The RT dose was 70 Gy in 35 fractions delivered by the conformal or IMRT technique. PFS was designated as the primary endpoint. Secondary endpoints included OS, LRC, Rate of distant metastasis (RDM) and acute and late toxicities. The median follow-up time was 2.8 years for the intervention arm and 2.6 years for the control arm. The PFS rate for 2 years was 36% in the intervention arm and 38% in the control arm, with a HR of 0.93 (95% CI, 0.73-1.20; P=0.58). LRC (HR, 0.98; 95% CI, 0.74-1.3; P=0.90) and OS (HR, 1.12; 95% CI, 0.86-1.46; P=0.39) also did not differ between the two arms. Distant metastasis was less frequent in the TPF arm (if first event, HR, 0.54, 95% CI, 0.30-0.99; P=0.05 in favor of TPF + cetuximab-RT arm; if first or later event, HR, 0.62 95% CI, 0.40-0.95; P=0.03 in favor of TPF + cetuximab-RT arm). These observations were found to be independent of p16 status (P-value for interaction, 0.35) (44). The PFS benefit of TPF + cetuximab + RT was found to be absent in both p16^+^ (HR, 0.78; 95% CI, 0.28-2.20; P=0.64) and p16^-^ (HR 1.28; 95% CI, 0.84-1.93; P=0.25) OPC. There was no difference in the rates of distant metastasis between p16^+^- and p16^-^-OPC. By contrast, a significant improvement in PFS was found in p16^+^-OPC compared with p16^-^-OPC (P<0.001), regardless of the treatment received. This is in accordance with the findings from previous research (45). In terms of adverse events, the TPF + cetuximab + RT arm experienced higher rates of grades 3 and 4 fever (9 vs. 0.6%; P<0.001), grade 3 and 4 neutropenia (26 vs. 6%; P<0.001) and febrile neutropenia (17 vs. 0%; P<0.001). However, the incidence of grade 3 and 4 skin reactions was higher in the cetuximab + RT arm (P<0.001). Based on these findings, TPF + cetuximab + RT was proposed to not be effective for improving the outcomes of patients with LAHNSCC compared with concurrent chemoradiotherapy.

The INTERCEPTOR-GONO study compared the effectiveness of induction chemotherapy followed by bio-RT (IBRT) against cisplatin based concurrent chemo-RT (CRT) in locally advanced head and neck cancers (29). The induction regimen was TPF, similar to that applied in the GORTEC 2007-02 study. The Bio-RT (BRT) consisted of cetuximab at a loading dose of 400 mg/m^2^ 1 week before RT, followed bs a reduced dose of 250 mg/m^2^ weekly for 7 weeks during RT. The RT dose was 70 Gy in 35 fractions delivered using IMRT or the three-dimensional conformal technique. The primary endpoint was OS. Secondary endpoints included PFS, objective response rate (ORR) and toxicities. The median OS was 59 months in both arms (HR, 1.05; 95% CI, 0.71-1.54; P=0.8). The median PFS was 31.6 months in the IBRT arm and 40.3 months in the chemoradiotherapy arm (HR, 1.03; 95% CI, 0.72-1.48; P=0.48). The ORR was 79% (95% CI, 0.55-0.72) in the intervention arm and 76% (95% CI, 0.55-0.73) in the control arm (P=0.47). Regarding adverse events, severe neutropenia (P=0.04) and skin toxicity (P=0.017) were significantly more common in the IBRT arm. Weight loss was significantly more frequent in the chemoradiotherapy arm (P=0.017). The findings of the INTERCEPTOR-GONO study supported those of the GORTEC 2007-02 study. Although patients in the GORTEC study were of advanced nodal disease resulting in worse outcomes, the findings from these two trials suggested the lack of effectiveness of three-drug induction chemotherapy followed by RT + anti-EGFR therapy compared to chemoradiotherapy alone in LAHNSCC (28,29,44).

### Induction chemotherapy followed by RT + anti-EGFR therapy vs. induction chemotherapy followed by chemoradiotherapy (1 RCT)

A previous open-label phase III trial conducted by The Spanish Cooperative Group for the Treatment of Head and Neck Cancer recruited patients with unresectable head and neck cancer who achieved stable disease following induction chemotherapy and randomized them into the cetuximab + RT (intervention arm) and cisplatin + RT (control arm) groups (30). Both arms received induction chemotherapy with the TPF regime. The RT dose was 70 Gy in 35 fractions delivered using the conformal technique. The concurrent agent was 100 mg/m^2^ cisplatin on days 1, 22 and 43 in the control arm, compared with 400 mg/m^2^ cetuximab on day 1 followed by 250 mg/m^2^ cetuximab weekly during RT. The primary endpoint assessed was the non-inferiority of the intervention arm compared with the control arm in terms of OS. Secondary endpoints included PFS, safety and QoL. The median follow-up duration was 43.9 months in the control arm and 41.1 months in the intervention arm. The median OS was 63.6 months in the control arm and 42.9 months in the intervention arm (HR, 1.106; 90% CI, 0.888-1.378; P=0.4492). The median PFS was 39.9 months in the control arm and 20.2 months in the intervention arm (HR, 1.190; 95% CI, 0.925-1.530; P=0.1759). A complete response or partial response was seen in 76.1% of the patients in the control arm and 79.7% of the patients in the intervention arm (P=0.3809). Acute AEs of special interest occurring in >10% of patients were mucosal inflammation (74.2%), radiation dermatitis (43.4%), dysphagia (28.3%), neutropenia (22.9%), anemia (18.5%), vomiting (17.6%) and ototoxicity (10.7%) in the control arm. In the intervention arm they included mucosal inflammation (79.7%), radiation dermatitis (46.5%), dysphagia (26.7%) and skin toxicity due to cetuximab (21.8%). Late AEs included neurotoxicity (11.2% cisplatin + RT arm vs. 4.0% in the cetuximab + RT arm; P=0.0058), xerostomia (22.0% in the cisplatin + RT arm vs. 27.7% in the cetuximab + RT arm; P=0.1777), and asthenia (5.9% in the cisplatin + RT arm vs. 5.9% in the cetuximab + RT arm; P=0.9703). Improvement of QoL dimensions was demonstrated in the cetuximab + RT arm compared with cisplatin + RT arm in terms of physical functioning (P=0.0287), appetite loss (P=0.0248), and social contact (P=0.0153).

Although there were positive findings in the cetuximab + RT arm in terms of QoL and AEs of special interest, the non-inferiority of cetuximab + RT over the standard treatment regimen of chemoradiotherapy (cisplatin + RT) in terms of OS could not be proven in this trial on patients with LAHNSCC who received prior induction chemotherapy.

### Anti-EGFR therapy in HPV^+^-OPCs (3 RCTs)

There were three randomized phase III trials for patients with HPV^+^-OPC that compared the efficacy of cisplatin + RT and cetuximab + RT. These were the De-ESCALaTE (31), NRG RTOG 1016(32) and TROG12.01(33) trials. The De-ESCALaTE trial included patients with advanced low-risk p16^+^ oropharyngeal SCC (31). Patients were randomly allocated to receive either cisplatin-based chemoradiotherapy or cetuximab bio-RT. The primary endpoint was overall (acute and late) severe toxicity (grades 3-4) and all grade toxicities. The incidence of overall grade 3-5 toxicities was similar between the two groups. The mean number of events per patient was 4.81 (95% CI, 4.23-5.40) for cisplatin and 4.82 (95% CI, 4.22-5.43) for cetuximab (P=0.98). The incidence of overall toxicity of all grades was also similar (mean number of events per patient, 30.1,95% CI,28.3-31.9 for the intervention arm and 29.2, 95% CI,27.3-31.0 in the control arm; P=0.49).

Secondary outcomes were OS, time to recurrence and QoL. The 2-year OS rate was 89.4% in the cetuximab group and 97.5% in the cisplatin group (HR, 5.0; 95% CI, 1.7-14.7; log-rank, P=0.0012). There was a significant improvement in the 2-year OS of patients at stage III (T4 or N3) who received cisplatin compared with that of patients who received cetuximab (93.3 vs. 67.1%; log-rank, P=0.0304). There was also a significant difference (HR, 4.4; 95% CI, 1.5-13.1; log-rank, P=0.0035) in the 2-year OS between the cisplatin (97.2%) and cetuximab (89.7%) groups in p16 and HPV DNA dual-positive patients. The 2-year recurrence rate was 16.1% in the cetuximab group, compared with 6% in the cisplatin group (HR, 3.4; 95% CI, 1.6-7.2; log-rank, P=0.0007). The mean global QoL scores on the European Organization for Research and Treatment of Cancer (EORTC) core QoL questionnaire (QLQ-C30) did not differ significantly between the two groups at any of the timepoints, with the mean difference at 24 months being 1.51 (in favor of cisplatin; P=0.09976) (46). There was a significant difference in social functioning (in favor of cetuximab; mean difference, 8.67; P=0.0374) at the end of treatment, although this difference disappeared 6 months later. In terms of swallowing, there was no significant difference between the two groups at 24 months (mean difference, 6.90 points in favor of cisplatin; P=0.1279).

Based on the findings of this trial the investigators concluded that cisplatin +RT should be considered as the standard of care in HPV+-OPC patients who are able to tolerate cisplatin (31,46).

The NRG RTOG 1016 trial was a non-inferiority trial in patients with low- and intermediate-risk HPV^+^-OPC at stages T1-T2, N2a-N3 M0 or T3-T4, N0-N3 M0(32). Patients were randomly allocated to either the RT + cetuximab or the RT + cisplatin groups. In this trial, the primary endpoint was OS, whilst secondary endpoints included PFS, LRF, DM, adverse events and QoL. The RT + cetuximab group did not reach the non-inferiority criteria for OS (HR, 1.45; one-sided 95% upper CI, 1.94; P-value for non-inferiority, 0.5056; one-sided log-rank, P=0.0163). The estimated OS at 5 years was 77.9% (95% CI, 73.4-82.5%) in the cetuximab group, compared with 84.6% (95% CI, 80.6-88.6%) in the cisplatin group. The PFS was significantly lower in the cetuximab group compared with the cisplatin group (HR, 1.72; 95% CI, 1.29-2.29; P=0.0002). The estimated 5-year PFS was 67.3% (95% CI, 62.4-72.2%) in the cetuximab group and 78.4% (95% CI, 73.8-83.0%) in the cisplatin group. LRF was found to be significantly higher in the cetuximab group (HR, 2.05; 95% CI, 1.35-3.10; P=0.0005). The estimated 5-year rates of locoregional failure were 17.3% (95% CI, 13.7-21.4%) in the cetuximab group and 9.9% (95% CI, 6.9-13.6%) in the cisplatin group. Regarding distant metastasis, there was no significant difference between the two groups (HR, 1.49; 95% CI, 0.94-2.36; P=0.09). The estimated 5-year rates of distant metastasis were 11.7% for the cetuximab group vs. 8.6% for cisplatin group. As regards QoL measurements, EORTC QLQ-H&N35 (European Organization for Research and Treatment of Cancer Quality of Life Questionnaire Head and Neck Module) completion patterns were similar between the two groups. Patient-reported severity scores of swallowing issues were found to be increased in both groups at the end of treatment compared with the pre-treatment scores. However, there was no statistically significant difference in the mean change of scores from baseline between the two groups (47.4±24.52 vs. 48.0±27.79; P=0.86). The proportions of grade 3-4 acute adverse events were similar in the cetuximab (77.4; 95% CI, 73.0-81.5%) and cisplatin groups (81.7; 95% CI,77.5-85.3%; P=0.1586). The late moderate to severe toxicity rate was 16.5% (95% CI, 12.9-20.7%) in the cetuximab group and 20.4% (95% CI, 16.4-24.8%) in the cisplatin group (P=0.1904). Acneiform rash was significantly more common in the cetuximab group, whereas the cisplatin arm experienced higher rates of myelosuppression, anemia, nausea, vomiting, anorexia, dehydration, hyponatremia, kidney injury and hearing impairment. Late hearing impairment was significantly more frequent in the cisplatin group. At treatment completion, 57.3% of patients (95% CI, 52.2-62.2%) in the cetuximab group and 61.5% of patients (95% CI, 56.5-66.3%) in the cisplatin group had a feeding tube. At 1 year after treatment, this proportion dropped to 8.4% (95% CI, 5.8-11.8%) in the cetuximab group and 9.2% (95% CI, 6.5-12.7%) in the cisplatin group (P=0.79). These findings suggested that cetuximab + RT was not superior to cisplatin + RT for improving the outcomes (in terms of OS or PFS) of patients with HPV^+^-OPC.

The TROG12.01 trial (33) included patients with low-risk p16^+^-OPC at stage III (excluding T1-2N1) or stage IV (excluding T4 and/or N3 and/or N2b-c if smoking history >10 pack years and/or distant metastases) according to the AJCC 7th edition guidelines. Patients were randomly allocated to receive IMRT (70 Gy in 35 fractions over 7 weeks) combined with either weekly cisplatin (40 mg/m^2^ for 7 cycles) or cetuximab (loading dose of 400 mg/m^2^ in the week before RT, followed by 250 mg/m^2^ weekly for 7 weeks along with RT). The primary endpoint of the study was symptom severity as assessed using the MDASI-HN from baseline to 13 weeks after RT completion. Secondary endpoints included other MDASI-HN scores, failure-free survival (FFS), OS, time to locoregional failure (TTLRF), pattern of failure, F-18 fluorodeoxyglucose (FDG)-positron emission tomography (PET) complete response rates at 13 weeks after RT, clinician-assessed acute and late toxicities, and rates of enteral feeding at 12 months. The median follow-up duration was 4.1 years (range 0.4-5.3 years). There was no statistically significant difference in the MDASI-HN symptom severity Area Under the Curve (AUC) from baseline to week 20 between the cetuximab and cisplatin arms (difference in AUC: 0.05 (95% CI, -0.19-0.30; P=0.66). There were no significant differences in other MDASI-HN scores, including modified symptom severity (P=0.97), symptom interference score (P=0.071), mucositis symptoms (P=0.91) or common symptoms (P=0.92). The 3-year FFS was superior in the cisplatin arm (93 vs. 80%; HR, 3.0; 95% CI, 1.2-7.7; P=0.015). There was no significant difference in 3-year OS between the groups (98% in the cisplatin group vs. 96% in the cetuximab group; HR, 2.3; 95% CI, 0.4-12.7; P=32) a The FDG-PET complete response rate at 20 weeks (13 weeks after the completion of RT) was 79% (95% CI, 69-87%) in the cisplatin arm and 69% (95% CI, 58-78%) in the cetuximab arm (P=0.16). Regarding acute toxicities, radiation dermatitis and acneiform rash were more common in the cetuximab arm, whereas febrile neutropenia, emesis, dry mouth, and fatigue were more common in the cisplatin arm. There was no significant difference in the incidence of late toxicities. No patient was on enteral feeding at 12 months after RT completion in either of the two arms. Freedom from distant failure was better in the cisplatin +RT arm compared to the cetuximab + RT arm (3-year freedom from distant failure rates were 97% vs. 88%, HR, 4.1; 95% CI, 1.2-14.9, P=0.018). These findings suggested that cetuximab + RT should remain as the SOC for patients with low-risk HPV-positive OPC.

## Discussion

A total of 12 phase III clinical trials in which patients received an anti-EGFR agent as treatment, either alone or in combination with RT and/or chemotherapy were reviewed in the present study. Only two studies (22,24) reported improvements in their respective primary outcomes. However, these two studies differed in terms of the nature of treatment combinations received and the primary outcomes evaluated. Bonner *et al* (22) revealed improved LRC (HR for locoregional progression/death, 0.68; P=0.005) when the intervention arm received cetuximab + RT, while the control group received RT alone (22,38). By contrast, in studies in which control patients received cisplatin + RT, the cetuximab group did not show any similar improvements in outcomes (23,26,28-33), where cetuximab + chemoradiotherapy (23) and cetuximab + RT (26,28-33) were not reported to be superior compared with standard chemoradiotherapy alone. Therefore, it was concluded that adding the anti-EGFR agent cetuximab to chemoradiotherapy confers no advantage over chemoradiotherapy alone for improving outcomes, where replacing cisplatin with cetuximab may even result in inferior survival outcomes for patients with LAHNSCC, including patients with HPV^+^-OPC (23,26,31-33).

Patil *et al* (24) reported significant improvements in PFS after the addition of nimotuzumab to chemoradiotherapy. The 2-year PFS was 61.8% (95% CI, 55.2-67.7) in the intervention arm and 50.1% (95% CI, 43.7-56.2) in the control arm (HR,0.69; 95% CI,0.53-0.89; P=0.004). with additional effectiveness in terms of LRC and DFS but not in terms of OS. The investigators who evaluated nimotuzumab have discussed the probable reasons for the positive outcomes based on the results of a subgroup analysis of the RTOG 0522 trial (23,24). This subgroup analysis of the RTOG 0522 trial revealed a trend towards improved outcomes in younger patients, patients with HPV negative tumors of the oropharynx or hypopharynx and patients in the T4 tumor subgroup (23). While these subgroups represented a small minority of the study population in the RTOG 0522 trial, the nimotuzumab trial had higher proportions of such patients (23,24). Moreover, a subgroup analysis of the Patil *et al* study also revealed that the addition of nimotuzumab led to a decrease in progression by 50% for p16 negative patients, strengthening their hypothesis (24). The weekly ‘lighter chemoradiotherapy’ regime, which resulted in fewer radiation interruptions compared with the RTOG 0522 trial, was also stated to be a probable reason for the positive outcomes in the nimotuzumab trial. Another possible reason is that nimotuzumab is biologically and structurally different from cetuximab and panitumumab in that it can interrupt both ligand-dependent and ligand-independent signaling of the EGFR pathway (24,47,48). However, before concluding that the combination of nimotuzumab + cisplatin + RT is superior to cisplatin + RT alone, it is imperative to consider that cisplatin was administered at a reduced dose (30 mg/m^2^ weekly) instead of 100 mg/m^2^ every 3 weeks or 40 mg/m^2^ weekly. Previous studies comparing cisplatin given at two different dose levels (30 mg/m^2^ weekly + RT vs. 100 mg/m^2^ every 3 weeks + RT) for the treatment of LAHNSCC have reported that 100 mg/m^2^ provided every 3 weeks is superior to 30 mg/m^2^ weekly in terms of LRC (49,50). Since it remains unclear if the efficacy of the chemoradiotherapy regimen was optimal in the study by Patil *et al* (24), it is currently inappropriate to make the assumption that adding nimotuzumab to the standard chemoradiotherapy procedure can confer any advantages over chemoradiotherapy alone (24). Larger clinical studies are required to test this hypothesis more adequately.

Phase III trials that tested the effectiveness of zalutumumab + chemoradiotherapy compared with chemoradiotherapy (5-year LRC, 70 vs. 74%; HR, 1.10; 95% CI, 0.81-1.50) or the effectiveness of panitumab + RT compared with chemoradiotherapy (2-year PFS: 76% (95% CI, 68-82) vs. 73% (95% CI, 65-79); HR, 0.95; 95% CI, 0.60-1.50; stratified log-rank test, P=0.83P=0.83 did not demonstrate any advantages of these anti-EGFR agents (25,27). Induction chemotherapy followed by bio-RT (cetuximab-RT) also did not confer any improvements in outcomes compared with concurrent chemoradiotherapy alone (28,29). In total, 6 of the 12 included studies reported QoL data (24,27,30-33). The trial by Hitt *et al* (30) found improvements in several QoL metrics, including physical functioning (P=0.0287), appetite loss (P=0.0248) and social contact (P=0.0153) in the cetuximab + RT group compared with the cisplatin + RT group. In the study by Patil *et al* (24), there was no difference the global health status QoL scores over time (P=0.396) between the nimotuzumab +chemoRT arm and the control arm (chemoRT) (51). Similarly, in the HN.6 trial (27,42), there was no difference in QoL between the panitumumab + RT and cisplatin + RT groups. The De-ESCALate (31,46), NRG RTOG 1016(32) and TROG 12.01(33) trials also found no difference in the QoL scores between the intervention and control groups.

Overall severe toxicity was assessed as the primary outcome in the De-ESCALaTE trial by Mehanna *et al* (31), revealing no significant difference between the cetuximab + RT arm and the cisplatin + RT arm. Symptom severity assessed using the MDASI-HN was set as the primary endpoint in the TROG 12.01 trial, but the trial did not reveal any significant differences between the cisplatin + RT and cetuximab + RT arms (33).

A number of trials included in the present review reported increased rates of mucositis (23-30) and skin reactions (22,23,25-31) following treatment with anti-EGFR agents. Three of the trials that used cetuximab have reported increased rates of acneiform rashes (22,26,33). Increased rates of neutropenia were reported in trials that used TPF + cetuximab + RT (28,29). By contrast, the incidence of nausea, vomiting, renal toxicity, and ototoxicity was higher following cisplatin administration (26,27,30,33).

Novel combination therapies, involving immunotherapy agents: the NRG-HN-004 trial tested the effectiveness of the immune checkpoint inhibitor durvalumab (an anti-programmed death-ligand 1 antibody) in combination with RT in cisplatin-ineligible patients compared to cetuximab +RT (52). The phase II results of this trial showed no improvement in PFS and significantly higher rates of locoregional failure with durvalumab +RT compared with cetuximab +RT (53). Furthermore, PembroRad is a phase II trial testing the efficacy of pembrolizumab (an anti-programmed cell death protein-1 antibody) compared with cetuximab concurrent with RT in patients with LAHNSCC who are refractory to cisplatin. However, the results of the study demonstrated that pembrolizumab concurrent with RT did not improve the tumor control rate or survival compared with cetuximab + RT, although there were less cases of toxicity in the pembrolizumab arm (54). REACH, a phase III trial, examined whether the combination of avelumab (an anti-programmed death-ligand 1 antibody) + cetuximab + RT is superior to cisplatin + RT, or cetuximab + RT, in cisplatin ineligible patients in terms of PFS. According to preliminary results, the avelumab + cetuximab + RT combination was tolerable for patients with LAHNSCC. However, further analysis showed no improvement in 1-year PFS with this combination compared to the control arm (55-57).

Of note, EGFR gene copy number alterations and high EGFR expression have been reported to be associated with poor prognosis in patients with HNSCC (58-60). However, to the best of our knowledge, at present, there are no validated molecular markers for the prediction of the response to anti-EGFR therapy (13) except for the finding that in patients who had skin rash when cetuximab was added to RT showed a positive trend in survival outcomes (13,38,39).

This systematic review had several limitations. Firstly, heterogeneity was found in the treatment combinations and the outcomes examined in the studies included in this review. Therefore, it was not possible to perform a meta-analysis. An analysis to test if any of the studies biased the results was not performed, which is a potential limitation of our review. Finally, in this review we included only phase III clinical trials and only 12 studies were eligible for inclusion. Therefore, evidence was synthesized based on the findings from these 12 studies published between 2006 and 2022.

In conclusion, cetuximab added to radical RT improved LRC, OS and PFS compared with RT alone, suggesting that cetuximab + RT is a treatment option for patients with LAHNSCC who cannot tolerate platinum-based chemotherapy (22,38,61). However, anti-EGFR monoclonal antibodies cetuximab, nimotuzumab, and zalutumumab when added to chemoradiotherapy did not improve OS compared to chemoradiotherapy alone (23-25). Induction chemotherapy followed by cetuximab + RT was not associated with any difference in outcomes compared with concurrent chemoradiotherapy (28,29). Phase III trials among p16^+^ patients with OPC reported inferior outcomes with cetuximab + RT compared with cisplatin + RT (31-33). Based on the findings of those studies, it is difficult to suggest that anti-EGFR therapy in any form confers any advantage over conventional chemoradiotherapy for p16^+^ patients with OPC. Results of the recent clinical trials evaluating novel combinations involving immunotherapy, against chemoradiotherapy for the treatment of patients diagnosed with LAHNSCC are not very encouraging (52-57). Additional studies in the field of translational biomarker research are required to build biomarker-based, novel treatment combinations of anti-EGFR agents for LAHNSCC.

## Supplementary Material

PubMed search results.

SCOPUS search on January 31, 2024

Embase search results (January 31, 2024)

Cochrane search results.

## Figures and Tables

**Table I:** RCTs testing the effectiveness of anti-EGFR monoclonal antibody in treating locally advanced head and neck squamous cell carcinoma.

A, RT + concurrent monoclonal anti-EGFR antibody vs. RT alone (1 RCT)									
Author/s, year	Anti-EGFR agent	Design	Population	Intervention	Control	Follow-up	Primary endpoint	Secondary endpoints	(Refs.)
Bonner *et al*, 2006, 2010	Cetuximab	RCT	Patients with locoregionally advanced head and neck cancer of oropharynx, hypopharynx, larynx (n=424)	High dose RT+ cetuximab High dose RT (Investigators were required to choose one of the following three RT regimens: 1.70.0 Gy in 35 fractions once daily, 2. 72.0-76.8 Gy in 60-64 fractions twice daily 3. 72.0 Gy in 42 fractions for concomitant boost schedule) + Weekly cetuximab administered 1 week before RT at a dose of 400 mg/m^2^, followed by 250 mg/m^2^ weekly for the duration of RT. (n=211)	High dose RT alone High dose RT (Investigators were required to choose one of the following three RT regimens: 1.70.0 Gy in 35 fractions once daily 2.72.0-76.8 Gy in 60-64 fractions twice daily 3. 72.0 Gy in 42 fractions for concomitant boost schedule; (n=213)	54 months	LRC locoregional control of disease duration: Interventio- 24.4 months; control- 14.9 months; HR for locoregional progression/death, 0.68 (95% CI, 0.52-0.89); P=0.005	OS: After a median follow-up duration of 54 months, the median OS was 49 months in the intervention arm compared with 29.3 months in the control arm; HR fordeath, 0.74 (95% CI, 0.57-0.97); P=0.03. PFS: Intervention-17.1 months; control-12.4 months; HR for disease progression/death, 0.70;95% CI, 0.54-0.90; P=0.006 ORR: Intervention-74%; control-64%; OR, 0.57 (95% CI, 0.36-0.90); P=0.02. Adverse effects (grade ≥3): No difference in terms of adverse events between the intervention and control arms except for acneiform rash and infusion reaction. Cetuximab arm had higher rates of acneiform rash (P=<0.001) and infusion reaction. (P=0.01)	(22,38)
B, Concurrent chemoradiotherapy + anti-EGFR monoclonal antibody vs. concurrent chemoradiotherapy alone (3 RCTs)									
Author/s, year	Anti-EGFR agent	Design	Population	Intervention	Control	Follow-up	Primary endpoint	Secondary endpoints	(Refs.)
Ang *et al*, 2014 (RTOG 0522)	Cetuximab	RCT	Patients with stage III or IV non-metastatic squamous cell carcinoma of the oropharynx, hypopharynx, or larynx Patients were stratified by tumour site (larynx vs. other), Nodal stage (N0 vs. N1-N2b vs. N2c-N3), Zubrod performance status (0 vs. 1), Use of IMRT (yes v no) and receipt of pre-treatment fused PET scan or CT scan (yes vs. No) (n=891)	Chemo-RT + cetuximab RT (72 Gy in 42 fractions over 6 weeks or 70 Gy in 35 fractions over 6 weeks using twice-a-day dosing once a week for 5 weeks) + cisplatin 100 mg/m^2^ D1 and D22 + Cetuximab (400 mg/m^2^ 1 week before RT and at 250 mg/m^2^ weekly during RT; Used IMRT or three- dimensional conformal RT (3DCRT) techniques for radiation planning and delivery (n=444)	Chemo-RT alone RT (72 Gy in 42 fractions over 6 weeks or 70 Gy in 35 fractions over 6 weeks using twice-a-day dosing once a week for 5 weeks) + cisplatin 100 mg/m^2^ D1 and D22; Used IMRT or three-dimensional conformal RT (3DCRT) techniques for radiation planning and delivery (n=447)	3.8 years	PFS The 3-year PFS was 58.9% (95% CI, 54.2-63.6%) in the intervention arm (cetuximab + cisplatin-RT) vs. 61.2% (95% CI, 56.7-65.8%) in the control arm (cisplatin-RT; P=0.76).	The 3-year OS was 75.8% (95% CI, 71.7-79.9%) in the intervention arm vs. 72.9% (95% CI, 68.7-77.1%) in the control arm (P=0.32). The 3-year LRF was 25.9% (95% CI, 21.7-30.1%) in the intervention arm compared with 19.9% (95% CI, 16.2-23.7%) in the control arm (P=0.97). The rates of distant metastasis in the two arms were not significantly different (13.0 vs. 9.7%; P=0.08). Adverse events: Patients who received cetuximab + cisplatin experienced more treatment- related grade 5 adverse events (10 vs. 3 events; P=0.05) compared with the control patients. The rate of grade 3 to grade 4 radiation mucositis was higher in the cetuximab arm compared with the control arm (43.2% vs. 33.3%). Intervention patients experienced higher rates of grade 3 to grade 4 skin reactions, fatigue, anorexia, and hypokalaemia during the first 90 days of therapy compared with the control patients.	(23,40)^a^
Patil *et al*, 2019	Nimotuzumab	RCT	Non-metastatic, stage III or IV SCC of oropharynx, larynx, hypopharynx, or oral cavity (n=536)	Chemo-RT plus + nimotuzumab High dose RT 5days/week, (gross tumour and lymph node disease received 70 Gy at 2 Gy/fraction, uninvolved nodal regions in the neck received 46-50 Gy. Other altered fractionation schedules were allowed if the biological equivalent dose for tmour control was similar to 70 Gy at 2 Gy/ fraction) 2D-RT, 3DCRT or IMRT + Cisplatin 30 mg/m^2^ weekly + Nimotuzumab 200 mg iv weekly (n=268)	Chemo-RT alone High dose RT 5days/week, (gross tumour and lymph node disease received 70 Gy at 2 Gy/fraction, uninvolved nodal regions in the neck received 46-50 Gy. Other altered fractionation schedules were allowed if the biological equivalent dose for tmour control was similar to 70 Gy at 2 Gy/fraction) 2D-RT, 3DCRT or IMRT + Cisplatin 30 mg/m^2^ weekly (n=268)	39.13 months	PFS The 2-year PFS were 61.8% (95% CI, 55.2-67.7) in the intervention arm and 50.1% (95% CI, 43.7-56.2) in the control arm (HR, 0.69; 95% CI, 0.53-0.89, P=0.004)	OS: 2-year OS, 63.8% (95% CI, 57.3-69.6) for intervention vs. 57.7% for control (95% CI, 50.9-63.6; P=0.163). LRC: 2-year LRC, 67.5% (95% CI, 60.9-73.3) for intervention vs. 57.6% (95% CI, 50.9-63.6) for control; HR, 0.67 (95% CI, 0.50-0.89; P=0.006). DFS: HR, 0.71; 95% CI, 0.55-0.92; P=0.008. Adverse events: Higher incidence of grade 3-5 mucositis in the nimotuzumab + cisplatin RT arm. The incidence of other grade 3-5 adverse effects was similar in the two groups. There was no difference the global health status QoL scores over time (P=0.396) between the nimotuzumab +chemoRT arm and the control arm (chemoRT)	(24,51)^a^
Eriksen *et al*, 2014, 2018 (DAHANCA 19)	Zalutumumab	RCT	Biopsy-verified HNSCC of the oral cavity, oropharynx, hypopharynx, and larynx (n=619)	Chemo-RT + zalutumumab Accelerated RT (66-68 Gy, 2 Gy/fraction, 6 fractions/week)+ nimorazole + weekly cisplatin 40 mg/m^2^ in stage III and IV + Zalutumumab 8 mg/kg weekly. Used IMRT (n=310)	Chemo-RT alone Accelerated RT (66-68 Gy,2Gy/ fraction,6 fractions/ week) + nimorazole + weekly cisplatin 40 mg/m^2^ in stage III and IV; Used IMRT (n=309)	36 months	LRC: 3-year LRC, 78% (intervention) vs. 79% (control) HR, 0.8; 95% CI, 0.6-1.2; 5-year LRC, 70.0% (intervention) vs. 74% (control) HR, 1.10; 95% CI, 0.81-1.50	Disease-specific survival (DSS; HR, 1.0; 95% CI, 0.7-1.7) and OS (HR, 0.9; 95% CI, 0.6-1.3) were similar in the intervention and control arms DSS: 3-year DSS; HR, 1; 95% CI, 0.7-1.7 5-year DSS; HR, 1.12; 95% CI, 0.79-1.60. OS: 3-year OS; HR, 0.9; 95% CI, 0.6-1.3 5-year OS; HR, 1.17; 95% CI, 0.89-1.52. Adverse events: Skin rash occurred in 94% of patients in the zalutumumab arm, (29% experienced grade 3-4 rash) and 13% of patients stopped zalutumumab due to skin rash. Patients in the zalutumumab arm experienced significant rates of confluent mucositis (70% vs. 56%, P=0.001) and grade 3-4 in-field skin reaction (27% vs. 4% P<0.0001)	(25,41)
C, RT + anti-EGFR monoclonal antibody vs. concurrent chemoradiotherapy alone (2 RCTs)									
Author/s, year	Anti-EGFR agent	Design	Population	Intervention	Control	Follow-up	Primary endpoint	Secondary endpoints	(Refs.)
Gebre-Medhin *et al*, 2021 (ARTSCAN III trial)	Cetuximab	RCT	Patients ≥18 years with previously untreated squamous cell carcinoma of oropharynx, hypopharynx or larynx, stage III-IV according to UICC TNM classification, 7th edition, without distant metastasis. Aimed for curative treatment with definitive RT (n=298)	RT +cetuximab RT (68 Gy to the primary tumour and lymph node metastases, 54.4 Gy to elective neck volumes given as daily fractions of 2.0 Gy and 1.6 Gy respectively, 5 days per week for seven weeks + Cetuximab (400 mg/m^2^ as loading dose 1 week before start of RT, followed by 250 mg/m^2^ per week for 7 weeks Patients with T3-T4 tumour underwent another random assignment of 63 Gy or 73.1 Gy given to the primary tumour as daily fractions of 2.15 Gy. Used IMRT, helical tomotherapy or volumetric arc therapy for radiation planning. (n=149)	Chemo-RT RT (68 Gy to the primary tumour and lymph node metastases, 54.4 Gy to elective neck volumes given as daily fractions of 2.0 Gy and 1.6 Gy respectively,5 days per week for seven weeks + Cisplatin 40 mg/m^2^ per week for seven weeks Patients with T3-T4 tumour underwent another random assignment of 63 Gy or 73.1 Gy given to the primary tumour as daily fractions of 2.15 Gy Used IMRT, helical tomotherapy or volumetric arc therapy for radiation planning. (n=149)	39 months	OS: 3-year OS, 78% (95% CI, 71-85) vs. 88% (95% CI, 83-94); adjusted HR, 1.63; 95% CI, 0.93-2.86; P=0.086. Post hoc subgroup analyses: HR for OS, 5.70 (95% CI, 1.67-19.5) for patients with p16^+^-OPC. Cetuximab + RT vs. cisplatin + RT (P=0.03)	LRC: Locoregional failure at 3 years, 23% (95% CI, 16-31) vs. 9% (95% CI, 4-14). Adjusted cause-specific HR, 2.49; 95% CI, 1.33-4.66; P=0.0045. Distant failure: NS. Adverse events: Acute mucositis (P=0.035), skin reactions (P=0.001) and acneiform rashes (P<0.001) were more common in the cetuximab group compared with the cisplatin group. Nausea (P=0.001), vomiting (P=0.015), acute kidney injury (P<0.001), tinnitus (P=0.002), dysphagia (P=0.033) and neutropenia (P<0.001) were significantly more common in the cisplatin arm. Late toxicities such as taste alteration and hearing impairment were more common in the cisplatin arm, while late pain and mucosal toxicities were more common in the cetuximab arm..	(26)
Siu *et al*, 2017 (HN.6 trial)	Panitumumab	RCT	Patients with locoregionally advanced SCC of the oral cavity, oropharynx larynx or hypopharynx (n=320)	Arm B RT+panitumumab Accelerated RT (70 Gy in 35 fractions over 6 weeks) + concurrent panitumumab (9 mg/kg) every 3 weeks, starting 1 week before RT (on days-7,15 and 36) Used IMRT or three-dimensional conformal RT (3DCRT) techniques for radiation planning and delivery (n=160)	Arm A Chemo-RT Standard fractionation RT (70 Gy 35 fractions over 7 weeks) + concurrent cisplatin 100 mg/m every 3 weeks (on days 1,22 and 43 of RT Used IMRT or three-dimensional conformal RT (3DCRT) techniques for radiation planning and delivery (n=160)	46 months	PFS 2-year PFS, 76% (95% CI, 68-82) vs. 73% (95% CI, 65-79); HR, 0.95; 95% CI, 0.60-1.50; stratified log-rank test, P=0.83P=0.83	OS: NS (arm B vs. arm A; HR, 0.89; 95% CI, 0.54-1.48; stratified log rank test P=0.66). The 2-year OS was 85% (95% CI, 78-90) for arm A, and 88% (95% CI, 82-92) for arm B. Recurrence: NS for 2-year cumulative incidence of local recurrence, regional recurrence, or distant recurrence. Adverse events: Ototoxic effects such as hearing loss and tinnitus, GI symptoms such as nausea, vomiting and dehydration, renal toxic effects, and weight loss were more common in the cisplatin + standard RT arm. Skin toxicity and grade ≥3 mucositis were more common in the accelerated RT + panitumumab group. The incidence of non- hematologic adverse events of grade ≥3 was similar in both arms (88% in arm A vs. 92% in arm B; P=0.25). There was no significant difference in the quality of life (QoL) parameters or swallowing outcomes between the two arms. At 1 year post treatment no difference was found between the arms in terms of FACT-H&N change from baseline: -1.70 (Control arm) and -4.81 (intervention), P=0.194 Swallowing related QOL (measured using SWAL-QOL and MDADI) declined from baseline to every subsequent time point.	(27,42)^a^
D, Three-drug induction chemotherapy followed by anti-EGFR monoclonal antibody + RT vs. chemoradiotherapy alone (2 RCTs)									
Author/s, year	Anti-EGFR agent	Design	Population	Intervention	Control	Follow-up	Primary endpoint	Secondary endpoints	(Refs.)
Geoffrois *et al*, 2018 (GORTEC 2007-02)	Cetuximab	RCT	Stage III to IV non-metastatic SCC of the oral cavity, oropharynx, hypopharynx, or larynx, with heavy nodal disease (N2b, N2c or N3), which was prone to distant metastasis (n=360)	TPF + cetuximab+ RT 3 cycles of TPF(docetaxel 75 mg/m2 on day 1+cisplatin 75 mg/m2 on day 1 + FU 750 mg/m2 on days 1-5) followed by cetuximab at a loading dose of 400 mg/m2 eight days before starting RT and a weekly dose of 250 mg/m^2^ for 7 weeks + RT RT dose was 70 Gy, in 35 fractions, 2 Gy per day, 5 days per week for 7 weeks delivered by 3-D conformal or IMRT technique. (n=181)	Chemo-RT 3 cycles of concurrent chemotherapy (carboplatin 70 mg/m^2^/day on days 1-4, and FU 600 mg/m^2^/day on days 1-4 continuous infusion every 3 weeks, during weeks 1,4 and 7 of RT) + RT. The RT dose was 70 Gy, in 35 fractions 2 Gy per day, 5 days per week for 7 weeks delivered by 3 D conformal or IMRT technique (n=179)	2.8 years for intervention arm, 2.6 years for the control arm	PFS: 2-year PFS, 0.36 (intervention) vs. 0.38 (control); HR, 0.93 (95% CI, 0.73-1.20; P=0.58)	OS: 2-year OS-NS; (HR, 1.12, 95% CI, 0.86-1.46; P=0.39). LRC: 2-year LRC-NS; (HR, 0.98, 95% CI, 0.74-1.3;P=0.90). RDM: If first event, HR, 0.54 (95% CI, 0.30-0.99; P=0.05) in favour of TPF + cetuximab-RT arm. If first or later event, HR, 0.62 (95% CI, 0.40-0.95; P=0.03) in favour of TPF + cetuximab- RT arm. Adverse events: The TPF + cetuximab-RT arm experienced higher rates of grades 3 and 4 fever (9% vs. 0.6%; P<0.001), grade 3 and 4 neutropenia (26% vs. 6%; P<0.001) and febrile neutropenia (17% vs. 0%; P<0.001). Grade 3 and 4 skin reactions were also more common in the cetuximab-RT arm (53% vs. 29%; P<0.001). Grade 3-4 mucositis was reported by 48% patients in the intervention arm and 50% patients in the control arm (p=0.7) Grade 3-4 skin reactions: 11 and 0% (outside RT fields) and 53 and 29% (inside RT fields) for the intervention and control arm, respectively (P=0.001); Grade 3-4 hypersensitivity to cetuximab occurred in 7% of patients (in the intervention arm); treatment- related deaths, 6.6 and 0.6% in intervention and control arm, respectively (P=0.0016).	(28,44)^a^
Merlano *et al*, 2020 (The INTERCEPTOR- GONO Study)	Cetuximab	RCT	Previously untreated stage III-IV, T1-4, N0-N3 histologically confirmed LASCCHN of oral cavity, oropharynx, larynx, hypopharynx Performance Status 0-1 (n=242)	Arm A(IBRT arm) TPF+ cetuximab- (loading dose, 400 mg/m^2^; 1 week before RT, then 250 mg/m^2^once a week for 7 weeks of RT +RT (70Gy 2Gy/day 5days/week) Used IMRT or three-dimensional conformal RT (3-DCRT) techniques for radiation planning and delivery (n=121)	Arm B (CRT arm) RT (70 Gy 2 Gy/day 5days/week; + concurrent cisplatin (100 mg/m^2^ every three weeks. Used IMRT or three-dimensional conformal RT (3-DCRT) techniques for radiation planning and delivery (n=121)	NA	OS: Median OS, 59 months in both arms; HR, 1.05 (95% CI, 0.71-1.54; P=0.8)	PFS: Median PFS, 31.6 (arm A) vs. 40.3 months (arm B); HR, 1.03 (95% CI, 0.72-1.48; P=0.48). ORR: 79% (95% CI, 0.55-0.72) in arm A, vs. 76% (95% CI, 0.55-0.73) in arm B, P=0.47. Adverse events: Severe neutropenia and skin toxicity were more common in the IBRT arm (P=0.04 and P=0.017, respectively). Weight loss was more common in the chemo-RT arm (P=0.017).	(29)
E, induction chemotherapy followed by RT + anti-EGFR monoclonal antibody vs. induction chemotherapy followed by chemoradiotherapy (1 RCT)									
Author/s, year	Anti-EGFR agent	Design	Population	Intervention	Control	Follow-up	Primary endpoint	Secondary endpoints	(Refs.)
Hitt *et al*, 2022	Cetuximab	RCT	Patients with unresectable locally advanced head and neck cancer received 3 cycles of TPF (docetaxel, cisplatin, and fluorouracil) (n=407)	Cetuximab (400 mg/m^2^ on day 1 followed by 250 mg/m^2^ cetuximab weekly during RT) + RT (conventionally fractionated form at 2 Gy/fraction for 35 fractions at 1 fraction per day; total dose was 70 Gy over 7 weeks. Used the 3-DCRT technique for all patients (n=202)	Cisplatin (100 mg/m^2^ on days 1, 22 and 43 of RT) + RT (conventionally fractionated form at 2 Gy/fraction for 35 fractions at 1 fraction per day; total dose was 70 Gy over 7 weeks. Used the 3-DCRT technique for all patients (n=205)	41.1 months (intervention) 43.9 months (control)	OS: Median OS, 63.6 (control) and 42.9 (intervention) months; HR, 1.106 (90% CI, 0.888-1.378; P=0.4492)	PFS: Median PFS, 39.9 (control) and 20.2 (intervention) months; HR, 1.190 (95% CI, 0.925-1.530; P=0.1759). ORR: Complete response or partial response, 76.1% (control) and 79.7% (intervention); P=0.3809. Adverse events: Rates of AEs of special interest occurring in >10% of patients in the control arm. were mucosal inflammation (74.2%), radiation dermatitis (43.4%), dysphagia (28.3%), neutropenia (22.9%), anemia (18.5%), vomiting (17.6%) and ototoxicity (10.7%) Rates of AEs of special interest occurring in >10% of patients in the intervention arm were: mucosal inflammation (79.7%), radiation dermatitis (46.5%), dysphagia (26.7%) and skin toxicity due to cetuximab (21.8%). Late AEs included neurotoxicity (11.2% cisplatin + RT arm vs. 4.0% in the cetuximab + RT arm; P=0.0058), xerostomia (22.0% in the cisplatin + RT arm vs. 27.7% in the cetuximab + RT arm; P=0.1777), and asthenia (5.9% in the cisplatin + RT arm vs. 5.9% in the cetuximab + RT arm; P=0.9703). Improvement of QoL dimensions was demonstrated in the cetuximab + RT arm compared with cisplatin + RT arm in terms of physical functioning (P=0.0287), appetite loss (P=0.0248), and social contact (P=0.0153).	(30)
F, Anti-EGFR therapy in HPV^+^-oropharyngeal cancer (3 RCTs)									
Author/s, year	Anti-EGFR agent	Design	Population	Intervention	Control	Follow-up	Primary endpoint	Secondary endpoints	(Refs.)
Mehanna *et al*, 2019 (De-ESCALaTE trial)	Cetuximab	RCT	Patients with HPV^+^ low-risk oropharyngeal cancer (non- smokers or life-time smokers with a smoking history of <10 years; n=334)	Cetuximab (400 mg/m² loading dose followed by weekly infusions of 250 mg/m² for seven weeks during RT) + RT(IMRT, 70 Gy,35 fractions) (n=168)	Cisplatin (100 mg/m² on days 1, 22 and 43 of RT) + RT (IMRT,70 Gy,35 fractions ) (n=166)	25.9 months	Overall severe toxicities, and all grade toxicities: Overall severe (grades 3-5) toxicity did not differ significantly between the groups. The mean number of events per patient was 4.81 (95% CI, 4.23-5.40) for cisplatin arm and 4.82 (95% CI, 4.22-5.43) for cetuximab arm (P=0.98). The incidence of overall toxicity of all grades was also similar (mean number of events per patient, 29.2, 95% CI, 27.3-31.0 in the cisplatin arm; and 30.1, 95% CI, 28.3-31.9 for the cetuximab arm P=0.49)	OS: 2-year OS, 89.4% in the cetuximab group vs. 97.5% in the cisplatin group; HR, 5.0 (95% CI, 1.7-14.7); log-rank P=0.0012. Recurrence rate: 2-year recurrence rate, 16.1% in the cetuximab group vs. 6% in the cisplatin group; HR, 3.4 95% CI, 1.6-7.2; log-rank P=0.0007. QoL: social functioning and swallowing: Ns for mean global QoL scores on EORTC QLQ-C30; did not differ significantly between the groups at any of the time points, mean difference at 24 months was 1.51 points (in favour of cisplatin; P=0.09976). Social functioning: At the end of treatment, there was a significant difference in social functioning in favour of cetuximab (mean difference, 8.67 points; P=0.0374; disappeared 6 months later). At 12 and 24 months, there was a significant difference in role functioning in favour of cisplatin (difference in mean scores of 8.3 points; P=0.0173). None of these differences reached the minimum for clinically meaningful difference of 10 points; Swallowing, Ns; at 24 months, mean difference, 6.90 points in favour of cisplatin; P=0.1279. Adverse events: Acute period severe short-term toxicities did not differ significantly; mean number of events/patient, 4.43 (95% CI, 3.84-4.97) for cisplatin arm and 4.35 (95% CI, 3.9-4.97; for cetuximab arm; P=0.84) Acute period rates of all grade toxicities were similar. Mean number of events/patient, 19.96 (95% CI, 18.8-21.1) in the cisplatin arm and 20.35 (95% CI, 19.18-21.52) in the cetuximab arm and (P=0.64). Late toxicity: rates of all-grade(P=0.49) late toxicities and severe (P=0.53) late toxicities were similar, but types of toxicities differed between the two groups. In the cetuximab group, the most common toxicity was gastrointestinal, mean number of events per patient: 1.9 (acute) and 0.2 (late), and higher rates of skin toxicity and infusion reactions. In the cisplatin group, the most common acute toxicity was gastrointestinal, mean number of events per patient: 2.12. The most common late toxicities were gastrointestinal (mean number of events/patient, 0.2), and labyrinthine (mean number of events/patient, 0.1). Haematological, metabolic and renal late toxicities were more in the cisplatin group than in the cetuximab group.	(31,46)^a^
Gillison *et al*, 2019 (NRG RTOG 1016),	Cetuximab	RCT	Patients with HPV^+^-OPC (n=805)	RT (Accelerated IMRT 70 Gy in 35 fractions over 6 weeks at 6 fractions/week) + cetuximab (400 mg/m² 5-7 days before RT initiation, followed by cetuximab 250 mg/m² weekly for 7 doses; n=399)	RT (Accelerated IMRT 70 Gy in 35 fractions over 6 weeks at 6 fractions/week) + cisplatin (100 mg/m² on days 1 and 22. (n=406)	4.5 years	OS: RT + cetuximab group did not reach the non-inferiority criteria for OS; HR, 1.45; one-sided 95% upper CI, 1.94. For non- inferiority, P=0.5056 and one-sided log- rank P=0.0163. Estimated OS at 5 years, 77.9% (95% CI, 73.4-82.5) in the cetuximab group vs. 84.6% (95% CI, 80.6-88.6) in the cisplatin group	PFS: PFS was significantly lower in the cetuximab group compared with the cisplatin group (HR, 1.72; 95% CI, 1.29-2.29; P=0.0002). 5-year PFS, 67.3% (95% CI, 62.4-72.2) vs. 78.4% (95% CI, 73.8-83.0). LRF and distant metastasis: The rate of LRF was significantly higher in the cetuximab group (HR, 2.05; 95% CI, 1.35-3.10; P=0.0005); 5-year proportions: 17.3% (95% CI, 13.7-21.4) vs. 9.9% (95% CI, 6.9-13.6). Distant metastasis, ns. No significant difference in distant metastasis between the two groups. Cetuximab vs. cisplatin HR, 1.49 (95% CI, 0.94-2.36; P=0.09). 5-year proportions: 11.7 vs. 8.6%. QoL: EORTC QLQ-H&N35 completion patterns were similar between the two groups. Patient-reported severity of swallowing issues was increased in both groups at the end of treatment compared with pre-treatment scores. However, between the groups, there was no statistically significant difference in the change of scores from baseline (mean, 47.4 vs. 48.0; P=0.86). At 1 year, the cetuximab group exhibited a significant increase in symptoms from pre-treatment levels compared with the cisplatin group although it was not a clinically important difference (7.6 vs. 2.5; P=0.0382). Adverse events: Proportions of acute moderate to severe toxicity: 77.4% (95% CI, 73.0-81.5) in the cetuximab group vs. 81.7% (95% CI, 77.5-85.3) in the cisplatin group; P=0.1586. Late moderate to severe toxicity: 16.5% (95% CI, 12.9-20.7) in the cetuximab group vs. 20.4% (95% CI, 16.4-24.8) in the cisplatin group; P=0.1904. Feeding tube issues, at treatment completion: 57.3% (95% CI, 52.2-62.2) of patients in the cetuximab group and 61.5% (95% CI, 56.5-66.3) of patients in the cisplatin group had a feeding tube. At 1 year after treatment, the proportions dropped to 8.4% (95% CI, 5.8-11.8) in the cetuximab group and 9.2% (95% CI, 6.5-12.7) in the cisplatin group (P=0.79).	(32)
Rischin *et al*, 2021 (TROG12.01 trial),	Cetuximab	RCT	Patients with low risk p16^+^- OPC AJCC 7th edition stage III (excluding T1-2N1) or stage IV (excluding T4 and/or N3 and/or N2b-c if smoking history >10 years and/or distant metastases; n=182)	Intensity modulated RT (70 Gy in 35 fractions over 7 weeks) with cetuximab with a loading dose of 400 mg/m^2^ in the week before RT, then 250 mg/m^2^ weekly for 7 doses (n=90)	Intensity modulated RT (70 Gy in 35 fractions over 7 weeks) weekly cisplatin (40 mg/m^2^ for 7 cycles; n=92)	4.1 years	Symptom severity: There was no difference in the MDASI-HN symptom severity AUC between the arms; difference in AUC between cetuximab- cisplatin was 0.05 (95% CI, -0.19-0.30; P=0.66)	FFS: The 3-year FFS was superior in the cisplatin arm (93 vs. 80%; HR, 3.0; 95% CI, 1.2-7.7; P=0.015). OS: There was no significant difference in 3-year OS; 98 and 96% (HR, 2.3; 95% CI, 0.4-12.7; P=0.32) for the cisplatin arm and the cetuximab arm, respectively. MDASI-HN scores: There were no significant differences in other MDASI-HN scores, including modified symptom severity (P=0.97), symptom interference score (P=0.071), mucositis symptoms (P=0.91) or common symptoms (P=0.92). FDG-PET complete response rates at 13 weeks post-RT: The FDG-PET complete response rate at 20 weeks (13 weeks after completion of RT) was 79% (95% CI, 69-87) in the cisplatin arm and 69% (95% CI, 58-78) in the cetuximab arm (P=0.16). Acute and late toxicities and rates of enteral feeding: Radiation dermatitis and acneiform rash were more common in the cetuximab arm, while febrile neutropenia, emesis, dry mouth and fatigue were more common in the cisplatin arm. There was no significant difference in late toxicities. No patient was on enteral feeding at 12 months in either of the two arms. Freedom from distant failure was better in the cisplatin +RT arm compared to the cetuximab+RT arm (3-year Freedom from Failure rates were 97% vs. 88%, HR, 4.1; 95% CI, 1.2-14.9 P=0.18)	(33)

^a^The second reference cited refers to an update of the same trial or another study associated with the same trial; this additional reference was added in order to provide further information. AEs, adverse events; AUC, area under the curve; BED, biological equivalent dose; CT, chemotherapy; DFS, disease-free survival; DSS, disease-specific survival; EORTC QLQ-C30, European Organization for Research and Treatment of Cancer Quality of Life Questionnaire, Core Module; QLQ-H&N35, European Organization for Research and Treatment of Cancer Quality of Life Questionnaire Head and Neck Module; FDG-PET, fludeoxyglucose-18 positron emission tomography; FFS, failure-free survival; FU, follow-up; GI, gastrointestinal; HPV, human papillomavirus; HR, hazard ratio; IBRT, induction chemotherapy followed by bio-radiotherapy; IMRT, intensity-modulated radiotherapy; iv, intravenous; LASCCHN, locally advanced head and neck squamous cell carcinoma; LRC, locoregional control; LRF, locoregional failure; MDASI-HN-MD, Anderson Symptom Inventory Head and Neck Symptom Severity Scale; NA, not applicable; NS, not significant; OPC, oropharyngeal carcinoma; OR, odds ratio; ORR, overall response rate; PFS, progression-free survival; QoL, quality of life; RCT, randomized clinical trial; RDM, rate of distant metastasis; RT, radiotherapy; SCC, squamous cell carcinoma; TPF, docetaxel, carboplatin and fluorouracil; UICC, Union for International Cancer Control.

## Data Availability

Not applicable.
