# Supplementary material for: Anti‑epidermal growth factor receptor monoclonal antibody therapy in locally advanced head and neck cancer: A systematic review of phase III clinical trials
Source: Med Int (Lond). 2024 May 29;4(4):41. doi: 10.3892/mi.2024.165 (PMC11170331; doi:10.3892/mi.2024.165)
Supplement: PubMed search results. [file Supplementary_Data.pdf]

Table SI. PubMed search results.

| Search | Query | Results | Time |
|---|---|---|---|
| #5 | Search: (((Head and neck squamous cell carcinoma) OR (locally advanced head and neck squamous cell carcinoma)) OR (head[Title/Abstract] AND neck cancer[Title/Abstract])) AND (((((cetuximab[Title/Abstract]) OR (panitumumab[Title/Abstract])) OR (zalutumumab[Title/Abstract])) OR (nimotuzumab[Title/Abstract])) OR (anti EGFR)) Filters: Randomized Controlled Trial | 128 | 22:53:28 |
| #4 | Search: (((Head and neck squamous cell carcinoma) OR (locally advanced head and neck squamous cell carcinoma)) OR (head[Title/Abstract] AND neck cancer[Title/Abstract])) AND (((((cetuximab[Title/Abstract]) OR (panitumumab[Title/Abstract])) OR (zalutumumab[Title/Abstract])) OR (nimotuzumab[Title/Abstract])) OR (anti EGFR)) | 2,213 | 22:50:20 |
| #3 | Search: ((((cetuximab[Title/Abstract]) OR (panitumumab[Title/Abstract])) OR (zalutumumab[Title/Abstract])) OR (nimotuzumab[Title/Abstract])) OR (anti EGFR) | 23,950 | 22:48:53 |
| #2 | Search: (((cetuximab[Title/Abstract]) OR (panitumumab[Title/Abstract])) OR (zalutumumab[Title/Abstract])) OR (nimotuzumab[Title/Abstract]) | 9,093 | 22:46:57 |
| #1 | Search: ((Head and neck squamous cell carcinoma) OR (locally advanced head and neck squamous cell carcinoma)) OR (head[Title/Abstract] AND neck cancer[Title/Abstract]) | 70,237 | 22:12:13 |

History and search details from January 31, 2024 showing entries 1 to 5 of 5 entries.

Table SII. SCOPUS search on January 31, 2024

| Search | Query | Results |
|---|---|---|
| #1 | (TITLE-ABS-KEY (head AND neck AND squamous AND cell AND carcinoma) OR TITLE-ABS-KEY (head AND neck AND cancer) OR TITLE-ABS-KEY (locally AND advanced AND head AND neck AND squamous AND cell AND carcinoma) | 121,849 |
| #2 | (TITLE-ABS-KEY (cetuximab) OR TITLE-ABS-KEY (panitumumab) OR TITLE-ABS-KEY (zalutumumab) OR TITLE-ABS-KEY (nimotuzumab) OR TITLE-ABS-KEY (anti AND egfr AND antibody)) | 34,016 |
| #3 | ((TITLE-ABS-KEY (cetuximab) OR TITLE-ABS-KEY (panitumumab) OR TITLE-ABS-KEY (zalutumumab) OR TITLE-ABS-KEY (nimotuzumab) OR TITLE-ABS-KEY (anti AND egfr AND antibody))) AND ((TITLE-ABS-KEY (head AND neck AND squamous AND cell AND carcinoma) OR TITLE-ABS-KEY (head AND neck AND cancer) OR TITLE-ABS-KEY (locally AND advanced AND head AND neck AND squamous AND cell AND carcinoma))) | 6,561 |
| #4 | TITLE-ABS-KEY (randomized AND controlled AND trials) | 1,066,474 |
| #5 | (((TITLE-ABS-KEY (cetuximab) OR TITLE-ABS-KEY (panitumumab) OR TITLE-ABS-KEY (zalutumumab) OR TITLE-ABS-KEY (nimotuzumab) OR TITLE-ABS-KEY (anti AND egfr AND antibody))) AND ((TITLE-ABS-KEY (head AND neck AND squamous AND cell AND carcinoma) OR TITLE-ABS-KEY (head AND neck AND cancer) OR TITLE-ABS-KEY (locally AND advanced AND head AND neck AND squamous AND cell AND carcinoma)))) AND (TITLE-ABS-KEY (randomized AND controlled AND trials)) | 767 |
| #6 | (((TITLE-ABS-KEY (cetuximab) OR TITLE-ABS-KEY (panitumumab) OR TITLE-ABS-KEY (zalutumumab) OR TITLE-ABS-KEY (nimotuzumab) OR TITLE-ABS-KEY (anti AND egfr AND antibody))) AND ((TITLE-ABS-KEY (head AND neck AND squamous AND cell AND carcinoma) OR TITLE-ABS-KEY (head AND neck AND cancer) OR TITLE-ABS-KEY (locally AND advanced AND head AND neck AND squamous AND cell AND carcinoma)))) AND (TITLE-ABS-KEY (randomized AND controlled AND trials)) AND (LIMIT-TO (DOCTYPE , "ar") OR LIMIT-TO (DOCTYPE , "cp")) | 397 |

.

Table SIII. Embase search results (January 31, 2024)

| Search | Query | Results |
|---|---|---|
| #4 | ((cetuximab:ti,ab,kw OR panitumumab:ti,ab,kw OR zalutumumab:ti,ab,kw OR nimotuzumab:ti,ab,kw OR 'anti egfr antibody':ti,ab,kw) AND (head AND neck AND squamous AND cell AND carcinoma OR (head:ti,ab,kw AND 'neck cancer':ti,ab,kw) OR ('locally advanced head':ti,ab,kw AND 'neck squamous cell carcinoma':ti,ab,kw))) AND 'phase 3 clinical trial'/de | 198 |
| #3 | (cetuximab:ti,ab,kw OR panitumumab:ti,ab,kw OR zalutumumab:ti,ab,kw OR nimotuzumab:ti,ab,kw OR 'anti egfr antibody':ti,ab,kw) AND (head AND neck AND squamous AND cell AND carcinoma OR (head:ti,ab,kw AND 'neck cancer':ti,ab,kw) OR ('locally advanced head':ti,ab,kw AND 'neck squamous cell carcinoma':ti,ab,kw)) | 4,067 |
| #2 | head AND neck AND squamous AND cell AND carcinoma OR (head:ti,ab,kw AND 'neck cancer':ti,ab,kw) OR ('locally advanced head':ti,ab,kw AND 'neck squamous cell carcinoma':ti,ab,kw) | 100,643 |
| #1 | cetuximab:ti,ab,kw OR panitumumab:ti,ab,kw OR zalutumumab:ti,ab,kw OR nimotuzumab:ti,ab,kw OR 'anti egfr antibody':ti,ab,kw | 18,827 |

Table SIV. Cochrane search results.

| Search | Query | Results |
|---|---|---|
| #1 | cetuximab | 2,674 |
| #2 | panitumumab | 826 |
| #3 | zalutumumab | 27 |
| #4 | nimotuzumab | 195 |
| #5 | anti egfr | 2,118 |
| #6 | #1 OR #2 OR #3 OR #4 OR #5 | 5,114 |
| #7 | head and neck squamous cell carcinoma | 3,767 |
| #8 | head and neck cancer | 8,719 |
| #9 | locally advanced head and neck squamous cell carcinoma | 950 |
| #10 | #7 OR #8 OR #9 | 9,636 |
| #11 | #6 AND #10 | 946 |
| #12 | phase 3 clinical trials | 15,2648 |
| #13 | #11 AND #12 | 418 |

Date run: January 31, 2024 at 16:53:16.
